# Impact of ERCC1, XPF and DNA Polymerase β Expression on Platinum Response in Patient-Derived Ovarian Cancer Xenografts

**DOI:** 10.3390/cancers12092398

**Published:** 2020-08-24

**Authors:** Federica Guffanti, Maria Francesca Alvisi, Elisa Caiola, Francesca Ricci, Marcella De Maglie, Sabina Soldati, Monica Ganzinelli, Alessandra Decio, Raffaella Giavazzi, Eliana Rulli, Giovanna Damia

**Affiliations:** 1 Laboratory of Molecular Pharmacology, Department of Oncology, Istituto di Ricerche Farmacologiche Mario Negri IRCCS, 20156 Milan, Italy; federica.guffanti@marionegri.it (F.G.); elisa.caiola@marionegri.it (E.C.); francesca.ricci@marionegri.it (F.R.); 2 Laboratory of Methodology for Clinical Research, Department of Oncology, Istituto di Ricerche Farmacologiche Mario Negri IRCCS, 20156 Milan, Italy; mariafrancesca.alvisi@marionegri.it (M.F.A.); eliana.rulli@marionegri.it (E.R.); 3 Mouse and Animal Pathology Lab (MAPLab), Filarete Foundation, Department of Veterinary Medicine, University of Milan, 20139 Milan, Italy; marcellademaglie@libero.it; 4 Department of Veterinary Pathology, University of Milan, 20133 Milan, Italy; sabina.soldati@bluewin.ch; 5 Unit of Thoracic Oncology, Medical Oncology Department, Fondazione IRCCS Istituto Nazionale dei Tumori, 20133 Milan, Italy; monica.ganzinelli@istitutotumori.mi.it; 6 Laboratory of Cancer Metastasis Therapeutics, Department of Oncology, Istituto di Ricerche Farmacologiche Mario Negri IRCCS, 20156 Milan, Italy; alessandra.decio@marionegri.it (A.D.); raffaella.giavazzi@marionegri.it (R.G.)

**Keywords:** ovarian carcinoma, patient-derived xenografts, cisplatin, nucleotide excision repair, base excision repair, biomarkers

## Abstract

Platinum resistance is an unmet medical need in ovarian carcinoma. Molecular biomarkers to predict the response to platinum-based therapy could allow patient stratification and alternative therapeutic strategies early in clinical management. Sensitivity and resistance to platinum therapy are partially determined by the tumor’s intrinsic DNA repair activities, including nucleotide excision repair (NER) and base excision repair (BER). We investigated the role of the NER proteins—ERCC1, XPF, ERCC1/XPF complex—and of the BER protein DNA polymerase β, as possible biomarkers of cisplatin (DDP) response in a platform of recently established patient-derived ovarian carcinoma xenografts (OC-PDXs). ERCC1 and DNA polymerase β protein expressions were measured by immunohistochemistry, the ERCC1/XPF foci number was detected by proximity ligation assay (PLA) and their mRNA levels by real-time PCR. We then correlated the proteins, gene expression and ERCC1/XPF complexes with OC-PDXs’ response to platinum. To the best of our knowledge, this is the first investigation of the role of the ERCC1/XPF complex, detected by PLA, in relation to the response to DDP in ovarian carcinoma. None of the proteins in the BER and NER pathways studied predicted platinum activity in this panel of OC-PDXs, nor did the ERCC1/XPF foci number. These results were partially explained by the experimental evidence that the ERCC1/XPF complex increases after DDP treatment and this possibly better associates with the cancer cells’ abilities to activate the NER pathway to repair platinum-induced damage than its basal level. Our findings highlight the need for DNA functional assays to predict the response to platinum-based therapy.

## 1. Introduction

Platinum-based therapy is one of the main effective treatments for different solid tumors [1]. The platinum-taxol doublet is first-line therapy for ovarian carcinoma with a high response rate (more than 80%). However, invariably, most patients relapse, with a resistant disease [2]. Understanding the mechanisms of platinum resistance is of paramount importance, as knowing a priori, that a tumor will probably not respond would help direct patients to an alternative therapy.

Cisplatin (DDP) and carboplatin are used in the management of ovarian carcinoma and their activity is mainly due to the formation of DNA monoadducts, adducts between adjacent purines (DNA intra-strand crosslinks) and between bases on opposite strands (DNA inter-strand crosslinks, ICLs). These DNA lesions, particularly the latter, are cytotoxic, as they interfere with transcription and replication, causing cell cycle arrest and inducing apoptosis [3,4].

The cell’s ability to cope with these lesions (i.e., its ability to repair the damage) is generally related to the drug’s cytotoxic effects. DDP-induced DNA lesions activate different DNA repair mechanisms depending on the cell cycle phase, the type and location of the lesions [1]. Even though there is ample preclinical evidence that nucleotide excision repair (NER), homologous recombination (HR), Fanconi Anemia (FA), base excision repair (BER), mismatch repair (MM) and translesion synthesis (TLS) are involved in the repair of DDP-induced DNA lesions, the exact mechanism is yet to be completely understood [3].

DDP monoadducts are preferentially repaired by BER and NER; inter-strand crosslinks by NER and the repair of ICLs involves the coordinated action of NER, HR and FA pathways. The key role of NER in removing DDP-induced DNA lesions has been suggested by the extreme sensitivity of cells lacking functional excision repair cross-complementation group 1 (ERCC1) and Xeroderma- Pigmentosum complementation group F (XPF) proteins [5]. The ERCC1 protein interacts with XPF to form a structure-specific endonuclease that cleaves DNA at 5′ near the site of DNA adducts, including platinum-DNA adducts, and is a limiting step in NER. The complex also has key roles in DNA recombination repair and ICLs repair [6].

This is why ERCC1 has been the most investigated potential biomarker of therapeutic response at the genomic level (analysis of single-nucleotide polymorphisms), at the transcriptional level (reverse transcriptase PCR) and at the protein level (immunohistochemistry- IHC) in different tumor types, in retrospective and prospective studies [7,8]. However, the results have been contrasting, with some studies showing—as expected—an inverse correlation between ERCC1 IHC-expression and therapeutic response [9,10], while others show no correlation [8,11]. These differences have been attributed to technical problems, not reproducible and the different performances of the antibodies used in the different studies [12], and to the existence of several ERCC1 isoforms with different functions. The *ERCC1* gene does, in fact, encode for four distinct variants (isoforms 201, 202, 203 and 204) derived from alternative splicing, and only isoform 202 can complex with XPF, forming the functional endonuclease involved in the NER pathway (ERCC1/XPF complex). This complex has been detected by proximity ligation assay (PLA) [13,14]; however, to our knowledge there are no studies on the expression of the ERCC1/XPF complex as a biomarker of DDP activity. The role of XPF has not been widely studied, though recent data from our laboratory showed that its mRNA levels positively associated with DDP sensitivity in patient-derived ovarian carcinoma xenografts (OC-PDX) [15].

DNA polymerase β (DNA pol β) is a key DNA polymerase in BER, whose major role is the repair of single base damage, generally resulting from oxidation and alkylation [16,17]. DNA pol β has also been involved in other DNA repair processes (HR, NER and non-homologous end joining- NHEJ) [18,19,20]. It has been reported to be mutated in 30% of human tumors and some of these mutations induce transformation and are associated with resistance to therapy [21,22,23]. In addition, its overexpression in tumors may be associated with increased DNA repair, increased mutation rate and resistance to DDP [24,25,26].

Preclinical validation of therapy response biomarkers requires representative preclinical pharmacologically characterized models [27,28,29] and, among them, OC-PDXs represent a valuable tool, as recently reported [30]. Given the importance of DNA repair in DDP cytotoxicity and having available an ovarian carcinoma PDX platform which, as a whole, represents the complexity of human ovarian carcinoma and has been pharmacologically characterized in vivo for the response to platinum-based therapy [31], we investigated the roles of the ERCC1/XPF complex and DNA pol β as possible biomarkers of DDP response.

## 2. Results

We have available in our laboratory a platform of patient-derived ovarian carcinoma xenografts (OC-PDXs) composed of both orthotopically (intraperitoneal/intraovary) and subcutaneously transplanted human ovarian carcinoma models. Some reports have highlighted a differential therapeutic response in preclinical models transplanted in different organ sites [32,33]. We have, however, reported that both the orthotopically and subcutaneously transplanted OC-PDXs of our xenobank have similar biological behavior and pharmacological response to therapy. In addition, these OC-PDXs have been shown to mimic the complexity and heterogeneity of human ovarian carcinoma, rendering them a suitable platform for biomarker studies [15,31]. We generated a tissue microarray (TMA) from PDX tumor samples, as described in the Material and Methods. The TMA includes 52 samples of stabilized OC-PDXs (41 high-grade serous/endometrioid carcinomas, 2 low grade serous/endometrioid carcinomas, 2 mucinous, 3 clear cell, 1 mixed müllerian, 1 carcinosarcoma and 2 undifferentiated carcinomas). Two core biopsies from distinct areas of tumor samples were selected by a pathologist and used to generate the TMA.

### 2.1. Relationship between ERCC1/XPF/DNA pol β mRNAs or Proteins and PLA-foci Expression in the Ovarian Xenobank.

Table 1 reports the descriptive analysis of ERCC1 and DNA pol β IHC, PLA foci numbers per nucleus and normalized ERCC1/XPF/DNA pol β mRNAs in all the OC-PDXs and in the subgroup of high-grade serous/endometrioid carcinomas. First, we analyzed the ERCC1 protein by IHC with the antibody anti-ERCC1 used in different studies [13,34]. Out of 52 samples, 49 displayed nuclear staining and, as already reported, some samples also showed cytoplasmic staining [35], lower than the nuclear stain (Figure S1). We considered only nuclear staining, since the cytoplasmic stain probably refers to ERCC1 isoform 203, not involved in the NER pathway [13]. We then tried different correlations, as ERCC1 and XPF are complexed and work jointly as endonucleases in different processes. The ERCC1 IHC-score did not correlate with the ERCC1 mRNA level, but it correlated positively with the XPF mRNA and ERCC1/XPF foci numbers when considering all the OC-PDXs (Spearman correlation coefficient 0.65, *p* < 0.0001 and Spearman correlation coefficient 0.29, *p* = 0.048, respectively, Table S1). Focusing on high-grade serous/endometrioid PDXs, as they account for 80% of ovarian carcinomas and the majority of our OC-PDX samples, only the correlation between the ERCC1 IHC-score with XPF mRNA was maintained (Spearman correlation coefficient 0.69, *p* = 0.0001, Table 2). The lack of correlation between ERCC1 IHC-score and ERCC1 mRNA might reflect the fact that the latter includes all the ERCC1 isoforms, while we focus only on nuclear ERCC1 IHC.

Using the PLA assay, we quantified nuclear ERCC1/XPF complexes in the OC-PDX samples (representative images are reported in Figure S2). As depicted in Table S2, the ERCC1/XPF foci were clear in all the samples, with no difference among tumor histotypes (Kruskal–Wallis *p* = 0.392). The ERCC1/XPF foci number per nucleus correlated with the ERCC1 IHC-score, and also with the DNA pol β IHC-score (Spearman correlation coefficient 0.58, *p* < 0.0001), when considering all the OC-PDXs (Table S1). When the same analysis was done in the high-grade serous/endometrioid carcinomas, the correlation with DNA pol β remained (Spearman correlation coefficient 0.62, *p* < 0.0001) (Table 2).

XPF mRNA and ERCC1 mRNA correlated with DNA pol β mRNA levels in all the OC-PDXs (Spearman correlation coefficient 0.40, *p* = 0.02 and Spearman correlation coefficient 0.32, *p* = 0.05, respectively; Table S1), but these correlations were not maintained in the high-grade serous/endometrioid OC-PDXs setting (Spearman correlation coefficient 0.36, *p* = 0.07 and Spearman correlation coefficient 0.24, *p* = 0.24, respectively; Table 2).

More than three quarters of the PDXs (78.4%) were positive for DNA pol β IHC (score ≥ 1) with a mean of 7.0 (standard deviation 3.4) (Table S2). There was no difference in DNA pol β staining among the histotypes (Kruskal–Wallis *p* = 0.315) and no correlation between protein expression and its mRNA level, when all the OC-PDXs were considered (Spearman correlation coefficient 0.17, *p* = 0.33; Table S1). Similar data were obtained in the high-grade serous/endometrioid OC-PDXs (Spearman correlation coefficient 0.17, *p* = 0.42, Table 2).

### 2.2. Correlation with Platinum Response

**Figure 1:**
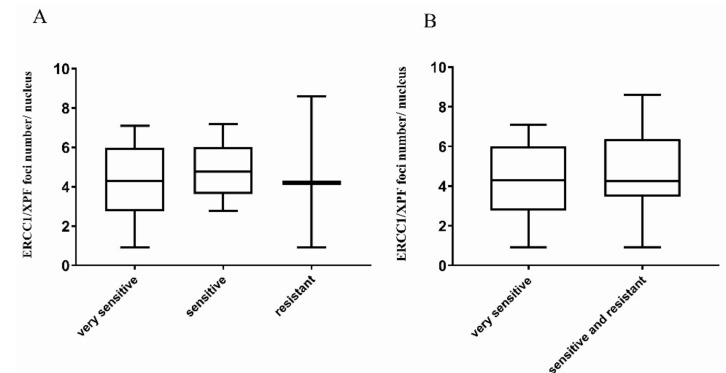
ERCC1/XPF foci number per nucleus in high-grade serous/endometrioid OC-PDXs. (**A**). The association between ERCC1/XPF foci number per nucleus and DDP-response in OC-PDXs very sensitive to DDP (10), sensitive (12), and resistant (3) is not statistically significant (Kruskal–Wallis *p* = 0.909). (**B**). The association between ERCC1/XPF foci number per nucleus in OC-PDXs very sensitive and sensitive/resistant to DDP is not statistically significant (Kruskal–Wallis *p* = 0.71). Data are mean ± standard deviation.

DDP activity data were available for 25 high-grade serous/endometrioid OC-PDX models (Table S2)—ten were very sensitive, 12 were sensitive and 3 were resistant to DDP. The ERCC1 IHC-score was not statistically related to sensitivity to DDP (Kruskal–Wallis *p* = 0.847, Figure S3). In addition, no differences were detected between the mean ERCC1/XPF foci numbers per nucleus and response to therapy, when considering the three response groups, very sensitive, sensitive and resistant (Kruskal–Wallis *p* = 0.909, Figure 1A) and the two response groups, very sensitive vs. sensitive/resistant (Kruskal–Wallis *p* = 0.71, Figure 1B).

**Figure 2:**
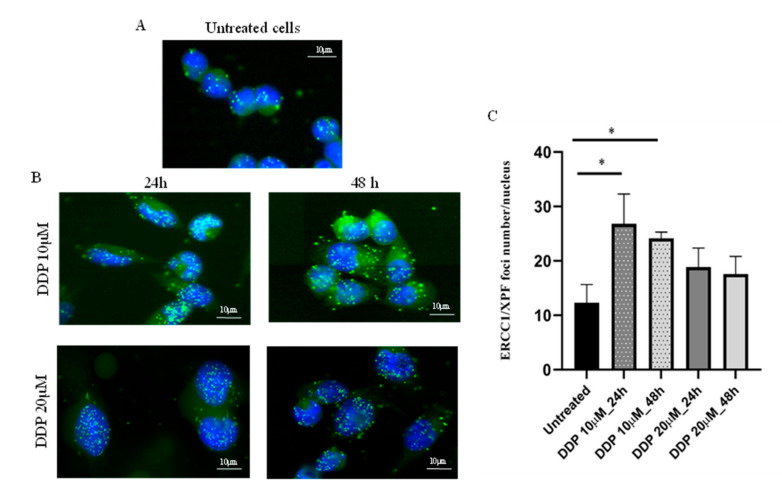
Detection and quantification of ERCC1/XPF foci numbers per nucleus (PLA) in A2780 ovarian cancer cells, untreated and after DDP. Nuclei of A2780 cells are shown in blue (DAPI) and ERCC1/XPF foci in the nuclei as green dots (FITC); magnification 40×, fluorescent microscopy. (**A**): ERCC1/XPF complexes expressed in untreated cells; (**B**): ERCC1/XPF foci in A2780 cells 24 h and 48 h after 10 μM (IC_50_) and 20 µM DDP-treatment; (**C**): quantification of ERCC1/XPF foci number per nucleus in A2780 untreated and DDP-treated cells with 10 µM (IC_50_) and 20 µM, fixed at two different time points. Each value is the mean ± standard deviation of three replicates and at least 50 nuclei were analyzed for each sample in different fields. Unpaired *t*-test was used to compare untreated vs. treated cells; * *p* ≤ 0.05.

These findings clearly suggest that our original hypothesis, that the basal level of ERCC1/XPF complex could be considered a read-out of functional NER, was not proved, as no correlation was found between the average ERCC1/XPF foci number per nucleus and DDP sensitivity. To clarify these unexpected results, we asked whether the ERCC1/XPF foci number per nucleus could be affected in tumor cells by DDP treatment. As depicted in Figure 2, ERCC1/XPF complexes were detected in untreated A2780 ovarian cancer cells and a significant increase in their number was observed at 24 h and 48 h after DDP treatment with 10 µM (*p* = 0.03 and *p* = 0.02, respectively). At the higher DDP dose of 20 µM, a trend towards an increased number of foci could be observed; however, no statistically significant difference was reached, likely due to the presence of a high number of apoptotic cells at both time points (*p* = 0.12 and *p* = 0.18, respectively).

**Figure 3:**
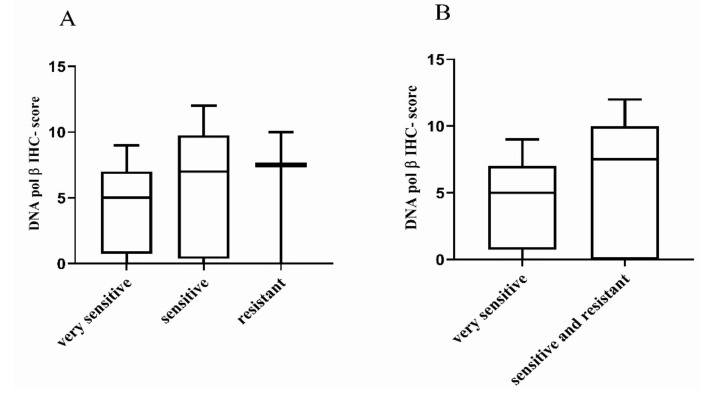
Distribution of DNA pol β IHC-score in high-grade serous/endometrioid OC-PDXs. (**A**). The association between DNA pol β protein expression in OC-PDXs very sensitive to DDP (*n* = 9), sensitive (*n* = 12), and resistant (*n* = 3) is not statistically significant (Kruskal–Wallis *p* = 0.664). (**B**). The association between DNA pol β protein expression in OC-PDXs very sensitive and sensitive/resistant to DDP is not statistically significant (Kruskal–Wallis *p* = −0.366). Data are mean ± standard deviation.

No difference between DDP sensitivity and the DNA pol β IHC-score (Kruskal–Wallis *p* = 0.664 and *p* = 0.366, respectively) was observed (Figure 3). Results were similar for DNA pol β mRNA expression level and the three categories of response (Kruskal–Wallis *p* = 0.204; Figure S4A), as already reported [15] and the two categories of response (Kruskal–Wallis *p* = 0.131; Figure S4B).

## 3. Discussion

DDP is one of the most effective chemotherapeutic agents for different tumor types. The platinum-taxol doublet is the first-line as adjuvant and neo-adjuvant chemotherapy in women with ovarian carcinoma with a high response rate (80%) [2]. However, most patients relapse, with a less DDP-responsive tumor [2]. This extreme sensitivity to DDP has been correlated with the fact that 50% of these tumors display a BRCAness phenotype due to inactivating mutations and/or promoter methylation in genes involved in HR [36]. Recently mutations and/or promoter hyper-methylation in NER genes have also been reported as associated with platinum response and at the basis of the different therapeutic responses to platinum and poly-ADP ribose polymerase inhibitors [37,38]. Not only do preclinical data strongly support the roles of HR and NER in the cytotoxic effect of DDP [5,39], but clinical data do too. Patients bearing tumors with functional inactivation of these pathways (HR and NER) experience lasting responses that translate to longer progression-free survival and overall survival than patients with proficient HR and NER DNA repair pathways [37,38].

These considerations have led to the proposal that increased DNA repair is associated with resistance to chemotherapy, and increased BER and NER activities have been reported in DDP resistant preclinical systems [5,18,40]. The clinical validation of these results has, however, been hampered by the choice of the biomarkers, the techniques used to validate the biomarkers (gene expression, protein expression, biochemical and functional assays) [7,12], the heterogeneity of cancer patients (Caucasian or Asian populations) and also tumor heterogeneity.

ERCC1 is the protein most investigated as a read-out of NER repair in different tumor types, including ovarian carcinoma, but with very contrasting results [41,42,43,44]. Stefensen et al. [43] reported how tumors with negative ERCC1 expression had responded more to platinum-based therapy than ERCC1-positive tumors and these findings were associated with significant differences in progression-free survival, in both univariate (*p* = 0.0012) and multivariate analysis (*p* = 0.006). However, no such positive correlation was found in other studies [42,45], even by the same authors [42,43].

The repair of platinum DNA damage relies on the coordinated interplay among different pathways. In the present manuscript we focused on the role of DNA pol β and ERCC1 in predicting platinum response in a platform of OC-PDXs, looking not only at their mRNA and protein levels, but also quantifying the functional complex ERCC1/XPF by PLA. No correlation was found between ERCC1 protein expression and DDP response in high-grade serous and endometrioid ovarian carcinomas. This lack of correlation between ERCC1 mRNA expression and the corresponding protein expression might explain the contrasting results on the predictive role of ERCC1 in response to DDP-based therapy in different tumor types, and further underlines the need for validated assays to identify predictive biomarkers.

We focused on the study of the ERCC1/XPF complex, which has been shown to be the active complex in the repair of DDP-induced DNA damage [12,13]. We did detect the complex in all the OC-PDX samples analyzed but found no correlation between DDP sensitivity and ERCC1/XPF foci number per nucleus. Possible explanations can be put forward for the lack of association we found. As we noted that the complex was up-regulated with DDP treatment in ovarian cells, we can hypothesize that is not the expression at the basal level of the complex that is the read-out of the NER activity, as originally thought, but rather its up-regulation upon DNA damage, similar to that reported for HR repair. It is the ability to increase RAD51 foci upon DNA damage that dictates the HR cell proficiency [46,47,48]. Higher ERCC1 protein levels in neo-adjuvant treated tumors compared to tumors of primary cyto-reductive surgery have been reported [49]; but even though these data suffer from the small sample size, they suggest an up-regulation of ERCC1 protein after DDP treatment. Another possible explanation is that NER, even if it is a key determinant in DDP induced DNA damage repair [5], it is not the only determinant, and other pathways (HR, Fanconi Anemia and translesion synthesis repair) are involved [50] and might justify the lack of correlation. Finally, a major role of tumor heterogeneity driven by genetic, epigenetic, and environmental inputs, directly linked to both innate and acquired resistance to therapy, including cytotoxic therapy, as recently reviewed [51], needs to be considered. Indeed, as for other cancers, ovarian carcinoma is also characterized by tumor heterogeneity caused by chromosomal instability, intratumor evolution, cellular plasticity, and multiple sources of stochastic variability. New single-cell sequencing technologies could lead to a high-resolution molecular phenotyping of large numbers of individual cancer cells, better addressing intratumor heterogeneity and its association with response to therapy [52].

BER has been reported to be involved in the repair of DDP-induced DNA lesion [3] and there is preclinical evidence suggesting a role of BER proteins in platinum’s cytotoxic effect [21,53,54]. The inactivation of proteins involved in BER makes tumor cells hypersensitive to DDP [3]. Enhanced DNA pol β expression and activity mediate replication, bypassing intra-strand crosslinks and is at the basis of the resistant phenotype in an ovarian cancer cell line resistant to DDP [55]. Recently, higher levels of DNA pol β were associated with DDP resistance, and the in vitro inhibition of DNA pol β with pamoic acid restored DDP sensitivity in KRAS (G12C) over-expressing non-small cell lung cancer (NSCLC) [40].

We already reported no correlation between DNA pol β mRNA and DDP response in our OC-PDXs platform [15]. Here, we show the lack of correlation between the DNA pol β protein level and DDP response. We found a strong correlation between DNA pol β protein levels and ERCC1/XPF foci number per nucleus (*p* < 0.0001). Since BER and NER pathways are both involved in the repair of DDP-induced monoadducts/intra-strand DNA lesions, this correlation suggests their coordination might cope better with the resolution of the damage.

There are a number of limitations in our study. The hypothesis that a single complex (ERCC1/XPF) and/or DNA pol β could be associated with platinum response can be viewed as simplistic and, as discussed above, other factors (i.e., HR proficiency, tumor heterogeneity) could play a more important role in platinum sensitivity/resistance. However, our aim was to find a biomarker that could be easily evaluated in the clinical setting by standardized techniques. Even if we tried to cope with the inter-xenograft (inter-patient) and intra-tumoral heterogeneity, as we used a TMA that includes two different distant pieces of the same tumor, and we verified the intra-tumor heterogeneity (i.e., both the distribution of ERCC1/XPF foci and DNA pol β in the two TMA samples were homogenous), the selected biomarkers were not strong enough to discriminate sensitive/resistant tumors. Ovarian carcinomas lack targetable genomic drivers, and at the same time, present unique and highly variable combinations of copy number aberrations and different cancer clones and the simple IHC analysis is likely insufficient to capture the complexity of ovarian carcinoma biology. More specific methodologies (i.e., single-cell multi-omics and parallel deconvolution of the mutational and epigenetic traits of individual cancer cells, as recently reviewed [51]) could better identify chemo-resistant subpopulations.

## 4. Materials and Methods

### 4.1. Patient-Derived Ovarian Carcinoma Xenobank

A platform of OC-PDXs from fresh tumor samples established in the last decade was available in our laboratory [15,31]. In total, 52 OC-PDXs were pharmacologically characterized for in vivo cisplatin (DDP) activity (Table S2). The majority of the OC-PDXs were high-grade serous/endometrioid ovarian carcinomas bearing a mutated *TP53*. DDP-antitumor activity has already been reported for most OC-PDXs [15,31].

All experiments involving animals were carried out using six- to eight-week old female NCr-nu/nu mice obtained from Envigo Laboratories (Bresso, Italy). Mice were maintained under specific pathogen-free conditions, housed in isolated, vented cages, and handled using aseptic procedures. The Istituto di Ricerche Farmacologiche Mario Negri IRCCS adheres to the principles set out in the following laws, regulations, and policies governing the care and use of laboratory animals: Italian Governing Law (D. lg 26/2014; Authorization n°.19/2008-A issued March 6, 2008 by Ministry of Health); Mario Negri Institutional Regulations and Policies providing internal authorization for persons conducting animal experiments (Quality Management System Certificate-UNI EN ISO 9001:2015–Reg, N°6121); the NIH Guide for the Care and Use of Laboratory Animals (2011 edition) and EU directive and guidelines (EEC Council Directive 2010/63/UE). All in vivo experiments complied with protocols approved by the Ethical Committee of the Istituto Mario Negri IRCCS and the Italian Ministry of Health (approval numbers 510-2016 and 296/2018-PR).

As already reported [31] in chemotherapeutic trials, mice with subcutaneously or intraperitoneally transplanted tumors were randomized to receive vehicle or DDP (from Sigma-Aldrich, St. Louis, MO, USA, prepared in saline just before use); the growth of subcutaneously xenografted tumors was measured twice a week with Vernier calipers, and tumor weights (mg = mm^3^) were calculated as follows: (length (mm) × width (mm)^2^)/2. The efficacy of the treatment was expressed as best tumor growth inhibition (T/C% = (median tumor weight of treated tumors/median tumor weight of control tumors) × 100). For intraperitoneal tumors, deriving from patient’s ascitic fluid, treatment efficacy was expressed as the increase in lifespan (ILS% (median survival day of treated tumors–median survival day of untreated tumors)/median survival day of untreated tumors) × 100). Drug activity was defined as follows: subcutaneous tumors were considered resistant with T/C ≥ 50%, sensitive with T/C 10–50% and very sensitive with T/C ≤ 10%; intraperitoneal tumors were considered resistant with ILS ≤ 40%, sensitive with ILS 40–100%, and very sensitive with ILS ≥ 100%, according to the published criteria [15,31]. Specimens were obtained from tumors growing subcutaneously (range 800–1200 gr) or from masses in the mouse peritoneal cavity at euthanasia, immediately snap-frozen and kept at −80 °C or fixed in 10% formalin, then paraffin-embedded (FFPE) until further analysis. FFPE tumor samples from the 52 ovarian PDXs were included in a tissue micro-array (TMA), using a standard technique [56].

### 4.2. DNA pol β and ERCC1 Immunohistochemistry (IHC) Expression in Ovarian OC-PDXs

DNA pol β and ERCC1 IHC was done in FFPE TMA 5 μm thick sections. Before IHC, the FFPE-TMA slides were de-paraffinized and boiled at 95 °C for 30 min in MS-Unmasker tris (EDTA) pH 7.8 solution (Diapath, Bergamo, Italy) for antigen-retrieval, then endogenous peroxidases were quenched and aspecific sites were blocked.

For DNA pol β IHC, the TMA slides were immune-stained with rabbit anti-DNA pol β primary antibody (Abcam, ab26343, Cambridge, United Kingdom) and incubated with a biotinylated secondary antibody (VC-BA-1000-MM15, Vector Laboratories, Burlingame, CA, USA). Sections were labelled by the avidin–biotin–peroxidase (ABC) procedure with a commercial immune-peroxidase kit (VC-PK-6100-KI01, Vector Laboratories). The immune-reaction was visualized with 3,3′-diaminobenzidine (DAB), (VC-SK-4100-KI01, Vector Laboratories) substrate. For ERCC1 IHC, a similar DAB-based immune-reaction was done. The TMA slides were incubated with anti-ERCC1 antibody (Santa Cruz Biotechnology, sc-10785, Dallas, TX, USA) diluted 1:100 in antibody diluent solution (Sigma Aldrich) overnight at 4 °C, then incubated with a secondary antibody Poly-HRP (RE7200-K, Novo Castra, Newcastle upon Tyne, UK). The immune reaction was visualized adding peroxidase 3,3′-diaminobenzidine (DAB) and its substrate (Novo Castra). Sections were then counter-stained with Mayer’s hematoxylin and cover slipped. Staining intensity was as follows: negative signal, 0; faint, 0.5; mild, 1; moderate, 2; marked, 3.

For DNA pol β IHC positive tumor cells, the score was 0 if none of the cells were positive for DNA pol β expression, 1 if the percentage of positive cells was between 0.1% and 25%, 2 if positive cells were between 26% and 50%, 3 between 51% and 75% and 4 between 76% and 100%. For ERCC1 IHC positive tumor cells, the score was 0 if no tumor cells were positive for ERCC1 expression, 0.1 if positive cells were between 1% to 9%, 0.5 between 10% and 49% and 1 ≥ 50%. The final IHC-score was obtained by multiplying the staining intensity for the score of positive tumor cells. The scores for both ERCC1 and DNA pol β were calculated as the mean of the two tumor scores in the TMA.

### 4.3. Gene Expression

Gene expression was measured as already described [15]. Briefly, tumor samples, with more than 80% of tumor cells, were homogenized in RNA lysis buffer in ice with an Ultra-Turrax (IKA, Staufen, Germany), and RNA was purified using the Maxwell 16 LEV SimplyRNA Cells kit (Promega, Madison, WI, USA). In all the samples by real time-PCR, the % of murine DNA contamination was established using primers specifically designed to distinguish human from murine *actin* and only samples with more than 85% human DNA were processed. Retro-transcription to cDNA was done using the High-Capacity cDNA Reverse Transcription kit (Thermo Fisher Scientific, Waltham, MA, USA). Genes selected were *DNA pol β*, *ERCC1* and *XPF*. Optimal primer pairs (Table S3) were chosen, spanning splice junctions, using Primer3 Input software (Primer3 Input. http://primer3.ut.ee/) and the human specificity was verified by detecting single-band amplicons of the PCR products. Absolute copy numbers of mRNA were determined by real time-PCR (ABI-7900, Applied Biosystems, Foster City, CA, USA) with the SYBR Green technique (Promega), using an EP Motion 5075 robot (Eppendorf, Hamburg, Germany). Standard curves for each gene were included for absolute quantification of mRNA.

Real time-PCR data were normalized using the geometric mean of two housekeeping genes, *actin* (*ACTB*) and *ciclophillin* (*CYPA*).

### 4.4. In Vitro Cell Culture and Drug Treatment

A2780 ovarian cancer cells were obtained from the American Type Culture Collection and had been authenticated by the authors in the last six months. The STR profiles were compared with the known American Type Culture Collection database. A2780 cells were maintained in RPMI supplemented with 1% L-glutamine and 10% fetal bovine serum (FBS). In total, 40,000 cells/mL were seeded in a 24-well plate. After 24 h, cells were treated with DDP at the IC50 10 μM and 20 µM for two hours. At the end of treatment, DDP was removed and fresh medium was placed in the wells; 24 h and 48 h after treatment, cells were fixed with pure cold methanol at −20 °C for 30 min, washed with PBS and kept in PBS at 4 °C until PLA.

### 4.5. Proximity Ligation Assay (PLA) for the ERCC1/XPF Complex Detection

FFPE-TMA 5 μm thick slices were put onto polylysine-coated glass slides. De-paraffination, antigen retrieval, peroxidase quenching and blocking were as described above for IHC. TMA slides were then co-incubated overnight at 4 °C with the two primary antibodies anti-ERCC1 (Santa Cruz biotechnology, sc-10785, Dallas, TX, USA) diluted 1:100 and anti-XPF (Thermo Scientific, MA5-12060, Waltham, MA, USA) diluted 1:200 in Antibody Diluent Solution (Sigma-Aldrich, St. Louis, MO, USA). The TMA-slides were then incubated with Duolink^®^ PLA Probes (Minus and Plus, Sigma-Aldrich, St. Louis, MO, USA) for the formation of oligonucleotides. The oligonucleotides were hybridized, ligated, amplified, and detected using Duolink^®^ Detection Reagents for Brightfield (Sigma-Aldrich). Slides were then counterstained with Nuclear Fast Red solution, dehydrated and mounted. Images were acquired with the VS120-Virtual Slide microscope (Olympus, Hamburg, Germany) at 40× magnification and processed with ImageJ software (Rasband, W.S., ImageJ, U.S. National Institutes of Health, Bethesda, MD, USA). Each nuclear dot corresponded to one ERCC1/XPF complex. The numbers of dots were normalized by the numbers of nuclei in the area of interest. At least 150 cancer cells were analyzed in each PDX core and at least three different areas per core were examined. Untreated and treated A2780 cells fixed on slides, after peroxidase quenching and blocking were washed with PBS and co-incubated overnight with primary antibodies, anti-ERCC1 and anti-XPF, and the processed as previously described, except for detection, which used Duolink^®^ In Situ Detection Reagents Green (Sigma-Aldrich). A2780 were incubated with DAPI solution for 10 min, washed with PBS then mounted.

PLA signals were visualized using the VS120-Virtual Slide microscope (Olympus) with FITC and DAPI filters at 40× magnification, and images were processed and analyzed as previously described.

### 4.6. Statistical Methods

Continuous variables were described, including the number of observations, mean, standard deviation (SD), median, inter-quartile range, minimum, maximum and number of missing values. Categorical variables were described with frequencies and percentages. Non-parametric analyses were done if the assumption of normality was not satisfied. Kruskal–Wallis tests were used to analyze the associations between ERCC1/XPF foci, ERCC1 IHC-score, DNA pol β-score, ERCC1/ XPF/DNA pol β mRNAs and response to DDP. Spearman correlation coefficients were calculated for the correlation between continuous variables.

Analyses were done, including all the OC-PDXs in the xenobank, and also in the group of high-grade serous/endometrioid PDXs. The response to DDP therapy was analyzed as a categorical variable according to two different classifications: in three groups (very sensitive, sensitive, resistant) or two (very sensitive vs. sensitive and resistant). This latter was dictated by preclinical evidence, suggesting that cells lacking ERCC1 were extremely sensitive to DDP (more than 100 times the ERCC1 of wild-type cells) [5]. A *p*-value ≤ 0.05 was considered significant.

Statistical analyses were done with SAS version 9.4 (SAS Institute, Cary, North Carolina). Unpaired *t*-test was used to analyze PLA foci numbers in untreated and DDP-treated samples, and *p* ≤ 0.05 was considered significant.

## 5. Conclusions

In conclusion, none of the proteins involved in the BER and NER pathways studied here predicted platinum activity in a panel of ovarian cancer xenografts. To our knowledge, this is the first report of the role of the ERCC1/XPF complex, detected by PLA, and the response to cisplatin in ovarian carcinomas. The results can be explained by the fact that the number of ERCC1/XPF complexes is further raised by DDP treatment, suggesting that it is not its basal level, but rather its increment that could predict the response to DDP. In this case, its clinical translatability is much harder as it would imply the availability of patient tumor samples treated with DDP in vivo or ex vivo. These results warrant further studies to investigate functional assays for NER and BER to predict the response to platinum-based therapy better, and also suggest that these pathways, even if important for the removal of DDP-induced DNA damage, and other factors—i.e., tumor heterogeneity with the presence of different cell clones- need to be taken into account.

## Figures and Tables

**Table 1:** ERCC1 and DNA pol β IHC-score, ERCC1/XPF foci number per nucleus (PLA) and ERCC1/XPF/DNA pol β gene expression levels in the OC-PDX population.

OC-PDXs	Descriptive Statistics	IHC-Score		PLA	Normalized Gene Expression Levels		
		*ERCC1*	*DNA pol β*	*ERCC1/XPF* *Foci Number*	*ERCC1*	*XPF*	*DNA pol β*
**All PDXs (*n* = 52)**	**Mean (SD)**	1.4(0.9)	5.5 (4.2)	5.0 (2.0)	1.1 (0.8)	0.0025 (0.0016)	0.0448 (0.0336)
	**Min–Max**	0.0–3.5	0.0–12.0	0.9–8.8	0.2–3.1	0.0005–0.009	0.004–0.1619
	**Missing**	3	1	3	16	16	16
**High-Grade PDXs (*n* = 41)**	**Mean (SD)**	1.2 (1.0)	5.3 (4.0)	5.1 (2.2)	1.2 (0.8)	0.0027 (0.0018)	0.0498 (0.0374)
	**Min–Max**	0.0–3.5	0.0–12.0	0.9–8.8	0.2–3.1	0.0009–0.0090	0.0040–0.1619
	**Missing**	1	1	2	2	2	2

Legend: *n*, number of OC-PDX samples; SD, standard deviation; Min–Max, range; PLA, proximity ligation assay.

**Table 2:** Correlations in the subgroup of high-grade serous/endometrioid OC-PDXs.

Method	Molecular Target	IHC-Score		PLA	Normalized Gene Expression Levels		
		*ERCC1*	*DNA pol β*	*ERCC1/XPF Foci Number*	*ERCC1*	*XPF*	*DNA pol β*
**IHC-Score**	**ERCC1**	1					
					
		39					
	**DNA pol β**	0.03	1				
		0.85					
		38	40				
**PLA**	**ERCC1/XPF Foci Number**	0.26	0.62	1			
		0.13	<0.0001				
		36	37	38			
**Normalized Gene Expression Levels by RT-PCR**	**ERCC1**	0.02	−0.07	0.15	1		
		0.94	0.72	0.47			
		25	25	25	26		
	**XPF**	0.69	−0.05	0.19	0.04	1	
		0.0001	0.83	0.36	0.83		
		25	25	25	26	26	
	**DNA pol β**	0.11	0.17	0.12	0.24	0.36	1
		0.62	0.42	0.58	0.24	0.07	
		25	25	25	26	26	26

Legend: In each box the first line reports the Spearman correlation index, the second line the *p*-value (in grey when significant), and the third the number of observations.

## Supplementary Materials

The following are available online at https://www.mdpi.com/2072-6694/12/9/2398/s1, Figure S1: ERCC1 immunohistochemical (IHC) expression on the TMA, Figure S2: ERCC1/XPF complexes detected by PLA on a FFPE OC-PDX’s tumor sample, Figure S3: ERCC1IHC-score in the high grade serous/endometrioid PDXs, Figure S4: DNA pol β mRNA in high grade serous and endometrioid PDXs, Table S1: Correlations found in all the OC-PDXs, Table S2: Tumor hystotypes, DDP sensitivity and raw data from IHC, PLA and gene expression analyses in the OC-PDX population under study, Table S3: List of the primers used in real time-PCR for the selected genes.
