# Supplementary material for: Impact of ERCC1, XPF and DNA Polymerase β Expression on Platinum Response in Patient-Derived Ovarian Cancer Xenografts
Source: Cancers (Basel). 2020 Aug 24;12(9):2398. doi: 10.3390/cancers12092398 (PMC7564949; doi:10.3390/cancers12092398)
Supplement: Supplementary file 1 [file cancers-12-02398-s001.pdf]

Supplementary Materials

Impact of ERCC1, XPF and DNA Polymerase β Expression on Platinum Response in Patient-Derived Ovarian Cancer Xenografts

Federica Guffanti, Maria Francesca Alvisi, Elisa Caiola, Francesca Ricci, Marcella De Maglie, Sabina Soldati, Monica Ganzinelli, Alessandra Decio, Raffaella Giavazzi, Eliana Rulli and Giovanna Damia

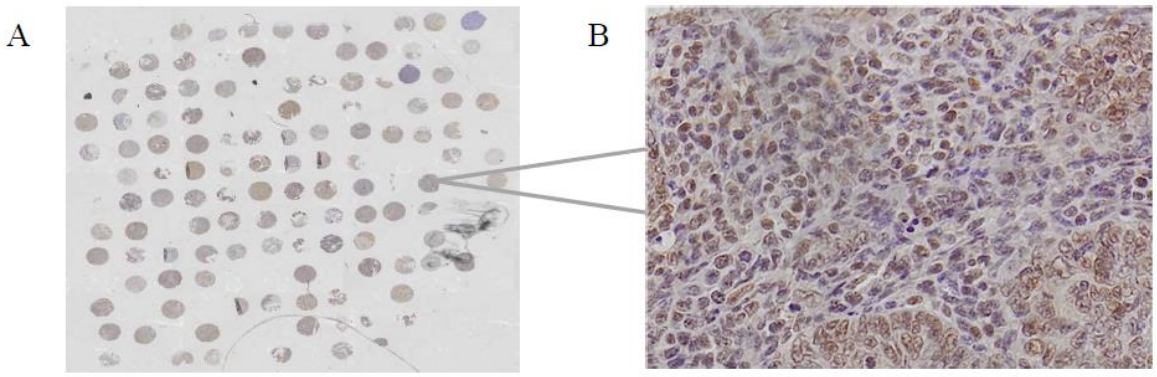

Figure S1. ERCC1 immunohistochemical (IHC) expression on the TMA. (**A**). Representative image of the FFPE-TMA comprehensive of 52 ovarian cancer PDXs, stained with antibody anti-ERCC1 (Santa Cruz Biotechnology, sc-10785, Dallas, TX, USA) magnification 2×. (**B**). Representative PDX#4, ERCC1-positive (IHC-score = 3.25). ERCC1-dots are present both in the cell nucleus than in the cytoplasm. Magnification 20×.

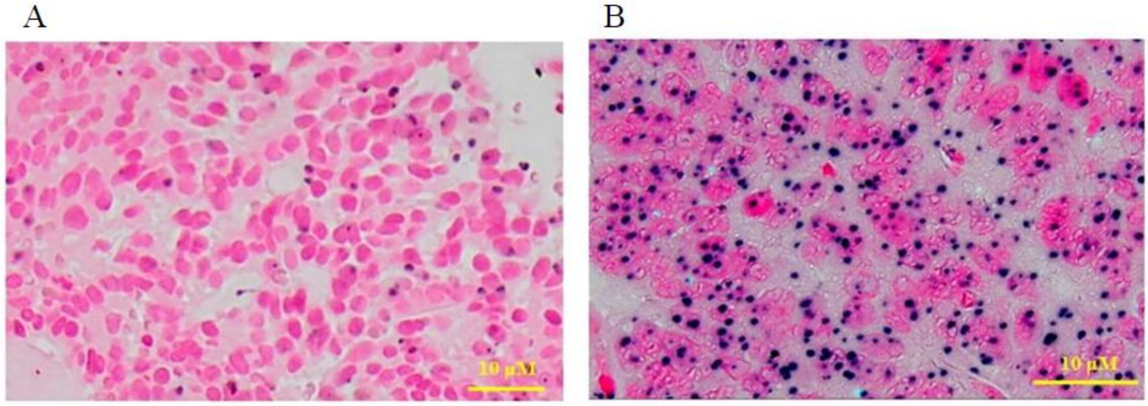

Figure S2. ERCC1/XPF complexes detected by PLA on a FFPE OC-PDX’s tumor sample. (**A**) Negative control. PLA was done without primary antibodies. It is possible to observe nuclei counterstained with Nuclear Fast Red (pink nuclei). (**B**) The same FFPE OC-PDX sample after PLA technique. ERCC1/XPF foci are visible as violet dots within epithelial cancer cells’ nuclei counterstained with Nuclear Fast Red (pink nuclei). Magnification 40×, brightfield microscopy.

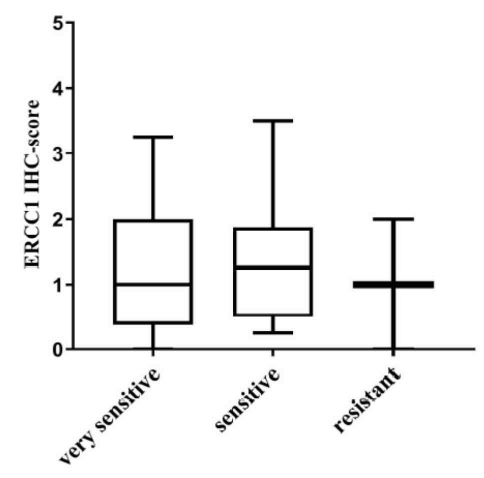

Figure S3. ERCC1-IHC score in the high grade serous/endometrioid PDXs. Distribution of ERCC1 protein expression in PDX very sensitive (*n* = 10), sensitive (*n* = 12) and resistant (*n* = 2) to DDP (see Material and Methods). Data are expressed as mean ± standard deviation.

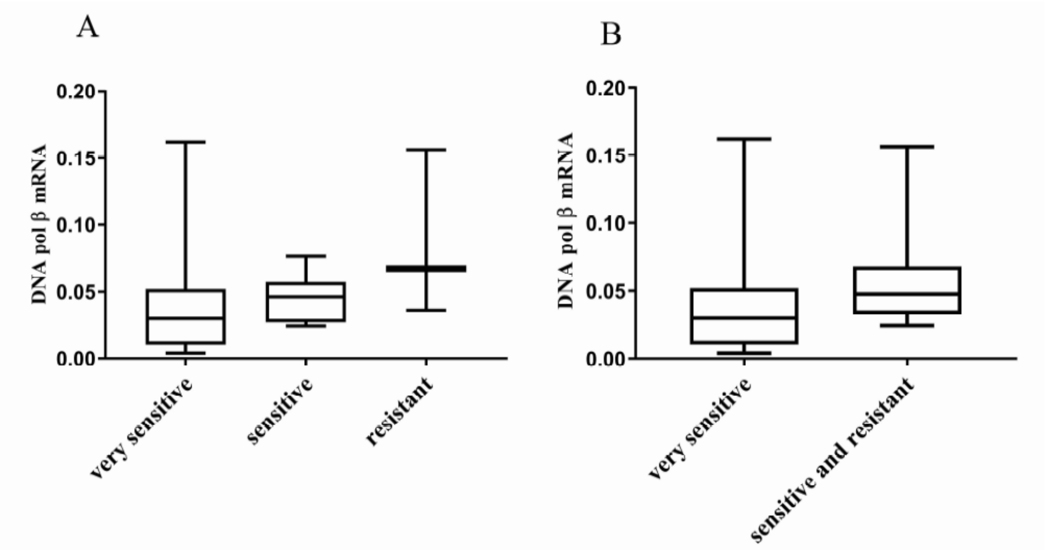

Figure S4. DNA pol β mRNA in high grade serous and endometrioid PDXs. (**A**). Distribution of DNA pol β mRNA in high grade PDXs very sensitive (*n* = 9), sensitive (*n* = 11) and resistant (*n* = 3) (see Material and Methods) to DDP. (**B**). Distribution of DNA pol β mRNA in very sensitive PDXs and sensitive/resistant to DDP. Data are expressed as mean ± standard deviation.

Table S1. Correlations found in all the OC-PDXs.

Method	Molecular Target	IHC-Score		PLA	Normalized Gene Expression Levels		
		ERCC1	*DNA pol β*	*ERCC1/XPF*	*ERCC1*	*XPF*	*DNA pol β*
**IHC-Score**	***ERCC1***	1	0.03	0.29	-0.11	0.65	0.14
			0.85	0.05	0.96	< 0.0001	0.43
		49	48	46	34	34	34
	DNA pol β	0.03	1	0.58	-0.11	0.11	0.17
		0.85		< 0.0001	0.53	0.54	0.33
		48	51	48	35	35	35
**PLA**	***ERCC1/XPF***	0.29	0.58	1	0.04	0.32	0.09
		0.05	< 0.0001		0.82	0.06	0.60
		46	48	49	35	35	35
**Normalized Gene Expression Levels by RT-PCR**	***ERCC1***	-0.11	-0.11	0.04	1	0.15	0.32
		0.96	0.53	0.82		0.40	0.05
		34	35	35	36	36	36
	XPF	0.65	0.11	0.32	0.15	1	0.40
		< 0.0001	0.54	0.06	0.40		0.02
		34	35	35	36	36	36
	DNA pol β	0.14	0.17	0.09	0.32	0.40	1
		0.43	0.33	0.60	0.05	0.02	
		34	35	35	36	36	36

Legend: in each box in the first line is reported the Spearman's correlation index, in the second line the p-value (in grey significant p-values), in the third line the number of observations.

Table S2. Tumor hystotypes, DDP sensitivity and raw data from IHC, PLA and gene expression analyses in the OC-PDX population under study.

					PLA	Normalized Gene Expression Levels (mRNA copies)		
**#ID PDX**	**Histotype**	**DDP Sensitivity**	**DNA pol β (IHC-score)**	**ERCC1 (IHC-score)**	**Mean (SD)**	**ERCC1**	**XPF**	**DNA pol β**
#PDX1	HG Serous	VS	9	2	7.090 (0.82)	3.0696	0.0029	0.0580
#PDX2	HG Serous	VS	8	0.5	6.079 (1.30)	0.5889	0.0009	0.0170
#PDX3	HG Serous	VS	4.5	0	5.752 (0.92)	2.1873	0.0013	0.1619
#PDX4	HG Serous	VS	0	3.25	3.685 (0.65)	0.5486	0.0026	0.0095
#PDX5	HG Serous	VS	1.5	1	3.267 (0.39)	1.5639	0.0011	0.0460
#PDX6	HG Serous	VS	6	2	4.891 (2.16)	-	-	-
#PDX7	HG Serous/Endom.	VS	0	0.5	0.925 (0.10)	0.7970	0.0018	0.0109
#PDX8	HG Endometrioid	VS	5	1	3.383 (0.74)	0.4336	0.0018	0.0040
#PDX9	HG Endometrioid	VS	-	0	1.250 (0.27)	1.4209	0.0010	0.0300
#PDX10	HG Endometrioid	VS	6	1	5.963 (1.04)	0.3397	0.0023	0.0400
#PDX11	HG Serous	S	8	1.5	7.171 (0.90)	0.8954	0.0038	0.0491
#PDX12	HG Serous	S	12	0.25	5.673 (1.06)	0.3985	0.0010	0.0551
#PDX13	HG Serous	S	1.5	3.5	3.173 (2.60)	0.8543	0.0055	0.0573
#PDX14	HG Serous	S	0	1.5	3.774 (0.44)	2.7480	0.0018	0.0462
#PDX15	HG Serous	S	9	1.5	4.152 (0.76)	0.2073	0.0019	0.0375
#PDX16	HG Serous	S	4.5	0.5	5.272 (1.00)	0.7744	0.0022	0.0244
#PDX17	HG Serous	S	10	0.5	7.039 (0.42)	0.7962	0.0013	0.0271
#PDX18	HG Serous	S	10.5	2	-	-	-	-
#PDX19	HG Endometrioid	S	8	1	4.243 (1.07)	0.4143	0.0040	0.0706
#PDX20	HG Endometrioid	S	0	0.5	2.779 (0.40)	1.5158	0.0014	0.0765
#PDX21	HG Endometrioid	S	0	2	-	0.8398	0.0038	0.0272
#PDX22	HG Endometrioid	S	6	0.75	5.408 (-)	1.2359	0.0019	0.0344
#PDX23	HG Serous	R	7.5	2	8.598 (1.95)	1.0627	0.0090	0.0673
#PDX24	HG Serous	R	0	0	4.202 (0.99)	2.9602	0.0030	0.0358
#PDX25	HG Serous	R	10	-	0.920 (0.16)	0.8152	0.0031	0.1563
#PDX26	HG Serous	-	12	3	6.941 (1.60)	3.071	0.0030	0.0336
#PDX27	HG Serous	-	10	2	8.765 (-)	1.5871	0.0033	0.0591
#PDX28	HG Serous	-	0	2	3.724 (0.68)	0.4129	0.0044	0.0590
#PDX29	HG Serous	-	0	-	3.245 (2.25)	-	-	-
#PDX30	HG Serous	-	7	2.5	6.309 (1.05)	-	-	-
#PDX31	HG Serous	-	7	1	1.306 (0.72)	-	-	-
#PDX32	HG Serous	-	4.5	2	6.063 (0.89)	-	-	-
#PDX33	HG Serous	-	9	1	6.572 (-)	-	-	-
#PDX34	HG Serous	-	5.5	2.5	7.377 (1.95)	-	-	-
#PDX35	HG Serous	-	7	0.5	5.928 (0.76)	-	-	-
#PDX36	HG Serous	-	10	2	6.260 (0.81)	-	-	-
#PDX37	HG Serous	-	12	2	8.597 (1.86)	-	-	-

#PDX38	HG Serous	-	12	0.75	-	-	-	-
#PDX39	HG Serous	-	2	1.25	4.535 (0.93)	-	-	-
#PDX40	HG Serous	-	12	0.75	8.732 (-)	-	-	-
#PDX41	HG Serous/Endom.	-	7.5	2	4.585 (1.03)	-	-	-
#PDX42	LG Serous	VS	8	0.75	3.964 (0.65)	1.0772	0.0029	0.0347
#PDX43	LG Endometrioid	S	0	0.25	3.165 (1.22)	0.6881	0.0005	0.0263
#PDX44	Mixed mullerian	S	1.5	0.5	3.146 (0.58)	0.9031	0.0011	0.0362
#PDX45	Carcinosarcoma	-	0	2.75	3.389 (1.10)	1.9701	0.0025	0.0653
#PDX46	Undifferentiated	-	3.5	2	5.899 (0.84)	-	-	-
#PDX47	Undifferentiated	S	3	2	4.453 (1.70)	0.8242	0.0019	0.0421
#PDX48	Mucinous	S	3	-	3.665 (0.81)	0.6693	0.0008	0.0089
#PDX49	Mucinous	R	12	2	5.069 (1.74)	0.7853	0.0030	0.0342
#PDX50	Clear cell	S	1.5	2.5	7.922 (-)	0.2010	0.0025	0.0252
#PDX51	Clear cell	S	0	1	5.318 (0.43)	1.8312	0.0028	0.0198
#PDX52	Clear cell	R	3.5	0.75	3.617 (1.85)	0.5060	0.0013	0.0250

-: data not available; VS: very sensitive (light grey box) to DDP; S: sensitive to DDP (grey); R: resistant to DDP (dark gray) as explained in Materials and Methods.

Table S3. List of the primers used in real time-PCR for the selected genes.

Function	Gene	Primer Forward	Primer Reverse
Base Excision repair (BER)	*DNA pol* β	TGCCTGGAGTAGGAACAAAAA	GGAAATTGATGGATGAACTCG
Nucleotide excision repair (NER)	*ERCC1*	CCAACAGCATCATTGTGAGC	TCTTGGCCCAGCACATAGTC
Nucleotide excision repair (NER)	*XPF*	TTGTGAGGAAACTGTATCTGTGG	AGCAAGCATGGTAGGTGTCA
Housekeeping	*ACT*β	TCACCCACACTGTGCCCATCTACGA	CAGCGGAACCGCTCATTGCCAATGG
Housekeeping	*CYPA*	GACCCAACACAAATGGTTCC	TTTCACTTTGCCAAACACCA

© 2020 by the authors. Licensee MDPI, Basel, Switzerland. This article is an open access article distributed under the terms and conditions of the Creative Commons Attribution (CC BY) license (http://creativecommons.org/licenses/by/4.0/).
